# Risk Factors Associated with Poor Physical Fitness in Three- to Six-Year-Old Children in Tujia-Nationality Settlement of China

**DOI:** 10.1155/2018/5702190

**Published:** 2018-11-08

**Authors:** Xiaoli Liu, Zheng Xiang, Chaoneng Liu, Xiangrong Shi, Xinfeng Yi, Mingyan Cheng, Hannah Schenck, Jonathan Bates

**Affiliations:** ^1^Department of Physical Education, Hubei University for Nationalities, Enshi, Hubei, China; ^2^Institute of Cardiovascular & Metabolic Disease, University of North Texas Health Science Center, Fort Worth, Texas, USA; ^3^National Stadium of Enshi Autonomous Prefecture, Enshi, Hubei, China; ^4^Yangqueba Primary School of Enshi Autonomous Prefecture, Hubei, China; ^5^Timberview Middle School, USA

## Abstract

**Background:**

Physical fitness has been recognized not only as an integrated predictor of the body's functional status, but also as an important marker of health outcomes. The aim of this study was to examine the factors associated with physical fitness among 3-6-year-old children within the Tujia-Nationality settlement in the years 2005, 2010, and 2014.

**Methods:**

Demographics questionnaires and fitness assessment were performed to identify the risk factors for poor physical fitness (PPF) among 3- to 6-year-old children in the years 2005, 2010, and 2014 in the area of southwest Hubei of China.

**Results:**

Of the 2128 children, 495 were classified as PPF (23.3%). In 2005, the percentage of PPF children was 21.7%, and the percentage of PPF children decreased from 29.1% in 2010 to 18.8% in 2014. Furthermore, Urban area children had a significant risk of PPF than rural area children (OR=1.299, P=0.031). Three-year-old children had 2.150-fold risk of PPF as compared to 6-year-old children. The children with less than 0.5 hours of activity time per day had 1.95-fold risk of PPF as compared to those with 1-2-hour activity time per day, respectively. Underweight and overweight/obese children had 2.74-fold and 1.67-fold risk of PPF as compared to normal weight children. Children had 1.97-fold risk of PPF when their father's schooling ceased after middle school and 1.51-fold risk of PPF when their father's schooling ceased after high school, respectively.

**Conclusions:**

These results demonstrated that the incidence of PPF children went up from 2005 to 2010 and then down from 2010 to 2014 within the Tujia settlement. For the children in this area, the risk factors associated with PPF included urban location, younger age, less than 1-hour activity time per day in kindergarten, underweight/overweight, low father's education level, and mother's childbearing age being less than 20 years.

## 1. Background

Physical fitness can be thought of as an integrated measure of most the body functions (skeletomuscular, cardiorespiratory, hematocirculatory, psychoneurological, and endocrine-metabolic) involved in the performance of daily physical activity and/or physical exercise [1, 2]. Improvements in cardiorespiratory fitness have been associated with a reduced risk of being overweight or obese in puberty, as well as improved cardiorespiratory health in adulthood [1, 3]. Tanaka et al. [4] reported that the physical fitness level had a strong inverse association with the incidence of all-cause mortality. Muscular strength has been positively associated with psychological health, and decreased all-cause premature mortality [5] and improvements in running speed (30 m sprint test) and explosive strength (vertical jump test) prove to be associated with the enhancement of bone mass in Spanish prepubertal boys [6]. Furthermore, poor coordination is associated with elevated levels of inattention and symptoms of anxiety and depression in childhood [7].

More so than the fitness level at any other stage of life, the level of physical fitness in childhood will have a marked effect on an individual's physical development and overall health in adolescence and adulthood [8, 9]. Thus, it seems that a priority for public health should be the monitoring of fitness, especially among the youngest, popularizing recommendations concerning daily exercise, and identifying risk factors which could hamper/harm/interfere with its natural development [10]. Some researchers focused on the physical fitness in children-adolescents, but there was less in preschool ages [1]. Moreover, there were no studies documented environmental, parental and postnatal feeding, and behavioral factors in relation to their physical fitness. The aim of the present study was to examine the factors that were associated with physical fitness in 3–6-year-old preschool children in the Tujia-Nationality settlement.

Physical fitness is partly genetically determined, but it is also strongly influenced by various environmental factors, among which physical activity is considered to be the most significant [9]. However, the given level of fitness of a child at a certain developmental stage is the result of the interaction between genetic and environmental factors [11]. These in turn may be influenced by perinatal factors, including but not limited to, gestational age, postnatal feeding, and maternal/paternal childbearing age [12]. Bouchard et al. found that environmental factors affect around 10-50% in the weight status of the children, while the genetics influence about 50-90% [13].

Meanwhile, the home environment is an important setting contributing to the physical activity behavior of children, which will influence the children's physical fitness [9, 14]. The relationship between socioeconomic status (SES) and physical fitness is closely correlated [15]. The Tujia-Nationality settlement is the most poverty stricken area in the southwest Hubei province in China; however, utilization of the highway and railway systems during the past 5 years has rapidly improved the local economy [16, 17]. While more families are beginning to realize the importance of a child's physical fitness, there still is some poor physical fitness (PPF) [18]. Therefore, we hypothesized that there should be a trend reversal in the children's PPF showed in this area from 2005 to 2014.

## 2. Methods and Participants

### 2.1. Participants

This study consisted of 3 cross-sectional surveys in the years 2005, 2010, and 2014, which was initiated and sponsored by the General Administration of Sport and the Ministry of Health of the People's Republic of China and supported and approved by the local government and the University for Nationalities in Enshi, Hubei province [19]. The study was designed to investigate the influencing factors of physical fitness in the southwest Hubei province of China. A total of 2,128 children aged 3- to 6-year-old boys and girls (n=568 in 2005, n=762 in 2010, and n=798 in 2014) whose parents voluntarily provided the informed consent participated in this study. The informed consent was reviewed and approved by the local ethical committee. All participants were recruited from the same Tujia settlement area in Hubei province of China.

### 2.2. Methods

The tests comprised two parts: a demographics questionnaire and a physical fitness assessment. Questionnaires inquired about each child's nationality, sex, geographical location (urban versus rural), age, gestational age, postnatal feeding method, activity time per day in kindergarten, parental education level, parental employment classification, parental childbearing age, and parental height/weight.

Physical fitness assessments included measurements for standing long jump, tennis ball throwing, sit-and-reach, turn back to run, walking on the balance beam, and continuous foot jump. Standing long jump tested using two-leg stance which was likely to show more reliable outcome of the muscle strength according to Cadenas-Sanchez et al. [6]. The children stood at a line marked on the ground with the feet slightly apart and then took off and landed using both feet, swinging the arms and bending the knees to provide forward drive, three tries, and using the best score. Tennis ball throwing was tested when the children stood at a line marked on the ground with the feet slightly apart and then threw the tennis ball with their best. Sit-and-reach tested the child's flexibility using a specially designed sit and reach equipment with a measuring scale; the children sat with bilateral knee straighten and then the hands pushed the cursor with their best. Turn back to run test required the children to* shuttle run* a 10-meter path, and the time was record. Walking on the balance beam tested the time of the children walking on a balance beam 3 meters long, 10 cm wide, and 30 cm high. Continuous foot jump required the children to jump over a soft package (10 cm long, 5 cm wide, and 5 cm high) placed on a horizontal line spaced every 0.5 meters between two lines on a 10-meter flat surface, using two-feet jump at the same time, jumping over one soft bag with one or two times, and skipping 10 soft squares in a row; the total time was recorded and reported [19]. These tests were considered valid and reliable for use with children [19].

The children performed all the tests in a gymnasium between July and August in the years 2005, 2010, and 2014. One parent of the study participants firstly completed a questionnaire. Then we measured the children's height, weight, standing long jump, tennis ball throwing, sit-and-reach, turn back to run, walking on the balance beam, and continuous foot jump accordance with the instructions in the manual of the General Administration of Sport, China [19]. The equipment used for fitness test recommended by the General Administration of Sport of China (made by the Shenzheng Hengkang Jiaye Limited Company, China) was suitable for preschool children and sensitive to distance/time which could be converted into a score. During the test, if the children cried or rejected test, we would take time and effort to make them comfortable and wait for them to take test again another day.

All physical fitness measurements and scoring criteria were classified according to the Citizen Physical Health Standard established by the Ministry of Education of China and the General Administration of Sport of China [19], which was published as an instruction book* Children Physical Health Standard*. According to the instruction in the manual of the General Administration of Sport, the test result of every component can convert into a numerical score, and the total score of the six components was added and reported [19]. When the children's score was less than 60% of the total score, they failed in the test. This assessment is considered valid and reliable for use with children [19].

BMI was calculated from weight (in kilograms)/height^2^ (in meter). BMI of the parents was classified into two levels: ≤25.0 kg/m^2^ as normal weight and >25.0 kg/m^2^ as overweight [20]. BMI of the children was categorized as underweight (≤15% BMI), normal weight (>15%,<85% BMI), and overweight (≥85% BMI) [21]. Overweight and obese groups were merged as one group in this study [21]. The gestational age was classified into three levels: premature (pregnancy ≥ 28, <37 weeks), full-term birth (pregnancy ≥ 37, ≤42 weeks), and postterm birth (pregnancy > 42 weeks) [22]. Postnatal feeding was defined as the feeding manner utilized during the first four months after the baby was born.

### 2.3. Statistical Analysis

All analyses were performed using the SPSS for Windows software package (version 18; SPSS Inc, Chicago, IL, USA). Descriptive statistics were obtained first; categorical variables were presented as the number of people (%). Chi square test was used to analyze the difference of unadjusted factors. Collinearity diagnostics were performed prior to further statistical analysis. A binary unconditional logistic regression model was used to analyze the potential risk factors (the first variable of every category in Table 3 as the control group). Potential influence factors included testing time, nationality, sex, geographical location (urban versus rural), age, gestational age, postnatal feeding, and activity time per day in kindergarten, parental education level, employment classification, childbearing age, and BMI. The dependent factor was “the result of physical fitness test score”. Retained methods used a forward stepwise approach. All variables significant at* P* < 0.05 were reserved in the final model.

## 3. Results

### 3.1. Children's Factors Connected with the Children's Physical Fitness

Of the 2,128 children, 495 failed in the physical fitness test, giving PPF children of 23.3%. The percentage of PPF children was 21.7% in 2005, and it increased to 29.1% in 2010, but it decreased to 18.8% in 2014 (Table 1), the difference of which was significant between 2005 and 2010 (P < 0.05) and between 2010 and 2014 (P < 0.05). The incidence of PPF children of Han nationality increased significantly compared to those of Tujia-Nationality (21.0% versus 25.9%) (*χ*^2^=7.124, P=0.009). There was no gender difference about the PPF children, and there were more PPF children in urban areas, but the difference was not significant. With age growth, the percentage of PPF children decreased significantly from 32.2% among 3-year-old children to 19.0% among 6-year-old children (3 yr versus 4 yr, 3 yr versus 5 yr, and 3 yr versus 6 yr, P < 0.05). The percentage of PPF was 25.5% in premature children, which decreased to 23.4% in full-term births and 16.9% in postterm births, though the difference was not significant (*χ*^2^ = 2.210, P = 0.331). Regarding postnatal feeding, 24.2% of the breastfed children, 21.4% of the milk fed children and 21.4% of the mixed fed children did not pass the physical test (all the differences were not significant, P > 0.05). The children's physical fitness improved as the daily activity time in kindergarten ranged between 1 and 2 hours. There were just 20.7% of children with normal weight who did not pass the physical test, which increased 34.5% (P < 0.05) with underweight children.

### 3.2. Parents' Characters Connected with the Children's Physical Fitness

The children's physical fitness was closely connected with parents' education, employment classification, childbearing age, and BMI [20] (Table 2). As the parents' education level increased, the children's physical fitness improved. When parental education levels were not higher than middle school, the percentage of PPF children was 27.8% (father's education) and 26.6% (mother's education), both of which decreased to 15.9% when the parents completed a college education, and the difference was significant (middle school versus college, P=0.001; high school versus college, P=0.001). The children had better physical fitness when the parents were labeled as white-collar workers, and the percentage of children's PPF increased when the employment classification of their parents was blue-collar (parents: white-collar workers versus blue-collar workers in rural, P < 0.05; fathers: blue-collar workers in urban versus blue-collar workers in rural, P < 0.05). Both parents' children bearing age affected children's physical fitness. When the father's childbearing age was over 35 years old, the percentage of PPF was 18.2%. However, when their childbearing age was between 20 and 35 and less than 20 years old, the percentage of children's PPF increased to 23.4% and 42.3% (<20yr versus 20-35 yr, <20yr versus ≥35 yr, P < 0.05), respectively. The mother's childbearing age had a similar trend as children's father. When the mother's childbearing age was less than 20 years, the percentage of PPF was 37.5%. However, when their childbearing age was between 20 and 35 and over 35 years, the percentage of children's PPF increased to 22.0% and 20.3% (<20 yr versus 20-35yr, <20yr versus ≥35 yr, P < 0.05), respectively. Parents' BMI was inversely related to the physical fitness of children: when the parents were overweight-obese, the percentage of PPF of children was less (fathers: normal versus overweight-obese, P=0.021).

### 3.3. Risk Factors Associated with Children's PPF in Binary Logistic Regression Analysis

Table 3 showed the risk factors of children's PPF, which were initially analyzed with time stratification. Since the risk factors that predicted the children's PPF were similar in the logistic regression models, the major predictors were merged together in the discussion for simplicity.

Compared to the data collected in 2014, there were more PPF children in 2005 and 2010, and the OR (odds ratio) values were 1.450 (CI= [1.081, 1.944], p=0.013) and 2.594(CI= [1.696, 2.837], p=0.001), respectively. Urban area children had a significant higher risk of PPF than rural area children (OR=1.299, CI= [1.024, 1.648], P=0.031). Age was inversely related to PPF, as 3-year-old children had 2.150-fold risk of PPF when compared to the 6-year-old children (CI= [1.566, 2.953], P=0.001). Activity time in kindergarten had a positive effect on children's physical fitness: children with less than 0.5 hours of activity time per day had 1.95-fold risk of PPF as compared to those with 1-2 hours of activity time per day (CI= [1.396, 2.723], P=0.001). Children with activity time between 0.5 and 1 hours per day had a 1.889-fold risk of PPF compared to those receiving 1-2 hours daily (CI= [1.330, 2.683], P=0.001). However, there was no fitness discrepancy between groups receiving 1-2 hours and those receiving more than 2 hours. Children's BMI was closely connected with physical fitness: underweight and overweight children had 2.741-fold (CI= [1.990, 3.773], P=0.001) and 1.674-fold (CI= [1.189, 2.356], P=0.0031) risk of PPF than normal weight children, respectively. Children had 1.973-fold (CI= [1.434, 2.714], P=0.001) risk of PPF when their father's schooling ceased after middle school, and 1.512-fold (CI= [1.113, 2.055], P=0.008) risk of PPF when their father's schooling ceased after high school, respectively. The mother's childbearing age proved to be a risk factor of a child's physical fitness: children whose mother's childbearing age was less than 20 years had 2.034-fold (CI= [1.435, 2.884], P=0.001) risk of PPF when compared to a maternal childbearing age between 20 and 35 years.

## 4. Discussion

The present study indicated that the incidence rate of PPF children went up from 2005 to 2010 and then down from 2010 to 2014 within the Tujia settlement in the Hubei province of China. For the children in this area, the main risk factors associated with PPF were found to be urban location, younger age, less than 1-hour activity time per day in kindergarten, underweight/overweight, low father's education level, and mother's childbearing age being less than 20 years.

Contrary to teenager‘s PPF in this area (because of the heavy load of study, they do not have the time to do exercise), [21] we found that the children with PPF went up from 2005 to 2010 and then down from 2010 to 2014, which was mainly attributed to parents' awareness of the importance of children's health [16, 23]. Parents have the ability to influence their child's physical activity by providing activity-related support. It also seemed to be associated with the economic improvement, which lead to an increase in physical activity facilities, particularly resources that were free to the public, which were consistent with previous observation [24]. In 2005, there were few public physical activity facilities, but in 2014, 2/3 of the communities offered free physical activity facilities [16]. Medical condition improved and all children had the opportunity to receive free physical examinations, which could screen for malnutrition and other common pediatric diseases. Meanwhile, the government offered a free nutrition plan [25], which was important for the poverty stricken children.

Because of the impact of environment conditions and economic status restrictions, rural children are more likely to take part in outdoor physical activity, are more independent, and possess better disease resistance [26]. Wang et al. found that the rural elementary school students performed better than urban children in bent-leg curl-ups and standing long jump tests in Taiwan, China [27]. Chillon et al. elaborated that rural Spanish children and adolescents had overall a healthier profile than their urban peers in terms of cardiorespiratory fitness, upper- and lower-limb muscular fitness, and adiposity, while they performed worse in speed-agility and flexibility [28]. From our study, we found that urban children had a significant risk of PPF compared to rural children.

We found 3-year-old children had a higher risk of PPF than 6-year-old children, though there was no statistical difference among 4- to 6-year-old children. The exact cause for the difference in PPF between 3- and 6-year-old was unknown. It might be that 3-year-old children are enrolled in kindergarten for the first year, so they do not adapt to the environment.

Physical activity is an important method for improving the health of children [29]. Physical activity reduces the risk for developing cardiovascular and metabolic diseases, body fat, and symptoms of anxiety and depression, while improving bone health and cardiorespiratory and muscular fitness in children [30]. Previous cross-sectional studies have reported that physical activity was positively associated with physical fitness in preschoolers, while a higher amount of self-reported physical activity has also been associated with better motor fitness [31]. This research found children taking part in more than 60 minutes of daily physical activity had less risk of PPF than their counterparts taking part in less than 60 minutes of daily physical activity. However, there was no significant difference between 1 and 2 hours and more than 2 hours per day, making 1-2 hours of daily activity adequate for maintaining physical fitness. This was consistent with the 2008 physical activity Guidelines for Americans and Europeans, which said all children and adolescents should perform at least 60 minutes of daily moderate-to-vigorous physical activity [32]. Therefore, kindergartens should increase children's physical activity by appropriate arrangements for sports and games, and the educational institutions should strengthen the supervision to ensure that children have physical activity per day in the kindergartens. The nondifference between 1-2 hours and >2 hours per day suggested that a threshold of adequate daily activity for preschool children to have a full benefit could take at least 1 hour but no more than 2 hours. Prolonged hours (>2 hours) would not add extra benefits for reducing PPF.

Both underweight and obesity are important factors affecting the level of physical fitness [33–35]. Monyeki et al. elaborated that underweight and obesity are reported to be related to decrease in physical exercise/work capability and then reduced health-related physical fitness, such as cardiorespiratory fitness [36] and strength and speed of movement [37]. Excessive fatness is found to be negatively associated with performance tasks in which the body is projected through space, as in standing broad jump and on tasks in which the body must be lifted in space, as in bent arm hang [37]. Artero et al. found overweight and obese adolescents presented a lower performance in 20 m shuttle run, bent arm hang, standing long jump, and shuttle run 4 x 10 m tests, but a higher performance in handgrip strength compared to normal weight [38]. Artero et al. also found that underweight was associated with a higher performance in the bent arm hang test in girls and a lower performance in handgrip in both genders compared to normal weight [38]. The present study indicated that, compared to normal weight children, underweight and overweight participants had a higher risk of PPF. This is likely due to the physical weakness of the underweight [39] and the physical inactivity of the overweight [40]. Our data suggested that not only overweight but also underweight seemed to be determinants of health-related fitness in preschoolers. Furthermore, in this preschool age group, the children with underweight seemed to have an elevated risk of PPF as compared to their overweight counterparts in terms of the odds ratios.

Parental education may influence physical activity, as parents serve as important behavioral role models from early childhood through to the teenage years [41]. Parents with higher levels of education are more likely to engage in health-promoting behavior [41]. Some studies showed a positive relationship between maternal education and juvenile physical activity [41]. Moreover, the educational background of parents is often used as a proxy for socioeconomic status. Our study found that the children had a higher risk of PPF when their fathers' education level was not higher than a middle school education and high school education, respectively.

In this study, we also found the mother's childbearing age affected children's physical fitness. Children had a higher risk of PPF with teenage childbearing mothers when compared to a maternal childbearing age between 20 and 35 years. Some researchers found the teenagers mothers were likely living in poverty [42], and they were too emotionally immature to be stable parents and not yet able to provide their children with the full range of support and stimulation they needed [43]. Shaw et al. found 14-year-old offspring of mothers who were aged 18 years and younger when they gave birth were more likely to have disturbed psychological behavior, poorer school performance, and poorer reading ability, were more likely to have been in contact with the criminal justice system, and were more likely to smoke regularly and to consume alcohol [43]. Our study further discovered the children with teenage mothers had a significant risk of PPF.

This study was among the first to discuss the risk factors of PPF about the 3-6-year-old children in this area. A large sample of preschooler youth was studied. We found 1-2 hours activity time per day in kindergarten was the best time for preschoolers. Despite this study's interesting findings, it had several limitations that should be acknowledged. First, we used the self-reported measurements about the parental BMI and the activity time per day in kindergarten. Second, we used the cross-sectional design. Third, people were randomly selected by our posted advertisements, so self-selection bias could not be avoided. In addition, future longitudinal study is needed which should include dietary data.

## 5. Conclusion

The incidence rate of PPF children went up from 2005 to 2010 and then down from 2010 to 2014 within the Tujia settlement in the Hubei province of China. For the children in this area, the main risk factors associated with PPF were found to be urban location, younger age, less than 1-hour activity time per day in kindergarten, underweight or overweight, low father's education level, and mother's childbearing age being less than 20 years. Further studies may examine the longitudinal impact of PPF on children's physical function and mental/cognitive development when they become graded students.

## Figures and Tables

**Table 1:** Factors associated with the children's physical fitness.

Variable/Category		Physical fitness assessment score		Total	Χ^2^	P
		Pass (%)	Fail (%)			
Testing time (yr)	2005	445(78.3)	123(21.7)	568^*∗*^	24.453	0.001
	2010	540(70.9)	222(29.1)	762^#^		
	2014	648(81.2)	150(18.8)	798		

Nationality	Han	733 (74.1)	256 (25.9)	989	7.124	0.009
	Tujia	900 (79.0)	239 (21.0)	1139		

Sex	Boys	827 (77.3)	243 (22.7)	1070	0.366	0.573
	Girls	806 (76.2)	252 (23.8)	1058		

Urban-rural	Urban	821 (76.6)	251 (23.4)	1072	0.028	0.878
	Rural	812 (76.9)	244 (23.1)	1056		

Age (yr)	3	351 (67.8)	167 (32.2)	518^*∗*#+^	32.072	0.001
	4	467 (78.2)	130 (21.8)	597		
	5	458 (80.1)	114 (19.9)	572		
	6	357 (81.0)	84 (19.0)	441		

Gestational age	Premature	73 (74.5)	25 (25.5)	98	2.210	0.331
	Full-term birth	1490 (76.6)	456 (23.4)	1946		
	Post-term birth	69 (83.1)	14 (16.9)	83		

Postnatal feeding	Breast feeding	1079 (75.8)	344 (24.2)	1423	1.959	0.375
	Milk	231 (78.6)	63 (21.4)	294		
	Mixed feeding	322 (78.5)	88 (21.5)	410		

Activity time per day in kindergarten	≤0.5 hs	195 (67.7)	93 (32.3)	288^*∗*#^	18.052	0.001
	0.5~1 hs	186 (70.7)	77 (29.3)	263^+&^		
	1.0~2.0 hs	653 (77.9)	185 (22.1)	838		
	>2.0	595 (81.0)	140 (19.0)	631		

Child's BMI^a^	Under weight	211 (65.5)	111 (34.5)	322^*∗*#^	28.058	0.001
	Normal weight	1178 (79.3)	308 (20.7)	1486		
	Over weight	243 (76.4)	58 (23.6)	318		

^a^BMI = body mass index; *∗*, the significant difference between 2005 and 2010, the significant difference of age between 3 and 4 years old, the significant difference of activity time per day in kindergarten between ≤0.5 hs and 1~2 hs, and the significant difference of child's BMI between underweight and normal weight; #, the significant difference of testing time between 2010 and 2014, the significant difference of age between 3 and 5 years old, the significant difference of activity time per day in kindergarten between ≤0.5 hs and >2.0 hs, and the significant difference of child's BMI between underweight and overweight; +, the significant difference of age between 3 and 6 years old and the significant difference of activity time per day in kindergarten between 0.5~1 hs and 1~2 hs; &, the significant difference of activity time per day in kindergarten between 0.5~1 hs and >2.0 hs and the significant difference of child's BMI between underweight and normal weight. All the categorized variables were made by using Chi-square test.

**Table 2:** Parental characters connected with the children's physical fitness.

Variable/Category		Physical fitness assessment score		Total	Χ^2^	P
		Pass (%)	Fail (%)			
Father's education	Middle school	604 (72.2)	232 (27.8)	836^*∗*^	24.124	0.001
	High school	622 (77.0)	186 (23.0)	808^#^		
	College	407 (84.1)	77 (15.9)	484		

Mother's education	Middle school	748 (73.4)	271 (26.6)	1019^*∗*^	17.157	0.001
	High school	600 (77.9)	170 (22.1)	770^#^		
	College	285 (84.1)	54 (15.9)	339		

Father's employment classification	White-collar workers	554 (79.5)	143 (20.5)	697^*∗*^	12.035	0.002
	Blue-collar workers in urban	684 (78.0)	193 (22.0)	877^#^		
	Blue-collar workers in rural	395 (71.6)	157 (28.4)	552		

Mother's employment classification	{1.00, Normal}...	320 (80.6)	77 (19.4)	397^*∗*^	8.808	0.012
	Blue-collar in urban	840 (77.6)	242 (22.4)	1082		
	Blue-collar in rural	471 (73.0)	174 (27.0)	645		

Father's BMI^a^	Normal	1279 (75.7)	411 (24.3)	1690	5.293	0.021
	Overweight-obese	348 (80.9)	82 (19.1)	430		

Mather's BMI	Normal	1565 (77.0)	468 (23.0)	2033	1.229	0.304
	Overweight-obese	64 (71.9)	25 (28.1)	89		

Father's child bearing age (yr)	<20	15 (57.7)	11 (42.3)	26^*∗*#^	7.696	0.021
	≥20,<35	1483 (76.6)	453 (23.4)	1936		
	≥35	135 (81.8)	30 (18.2)	165		

Mother's child bearing age (yr)	<20	110 (62.5)	66 (37.5)	176^*∗*#^	21.937	0.001
	≥20,<35	1474 (78.0)	416 (22.0)	1890		
	≥35	47 (79.7)	12 (20.3)	59		

^a^BMI = body mass index; *∗*, the significant difference of father's education between middle school and college, the significant difference of mother's education between middle school and college, the significant difference of father's employment classification between white-collar worker and blue-collar workers in rural, the significant difference of mother's employment classification between white-collar worker and blue-collar workers in rural, the significant difference of father's child bearing age between less than 20 years and 20~35 years, and the significant difference of mother's child bearing age between less than 20 years and 20~35 years; ^#^the significant difference of father's education between high school and college, the significant difference of mother's education between high school and college, the significant difference of father's employment classification between blue-collar workers in urban and blue-collar workers in rural, the significant difference of father's child bearing age between less than 20 years and more than 35 years, and the significant difference of mother's child bearing age between less than 20 years and more than 35 years. All the categorized variables were made by using Chi-square test.

**Table 3:** Risk factors associated with children's poor physical fitness in binary logistic regression analysis.

Variable/Category		B	Wals	Sig.	Exp (B)	95% C.I.	
						Down	Up
Testing time (yr)	2014	1					
	2005	.372	6.158	.013	1.450	1.081	1.944
	2010	.786	35.845	.000	2.194	1.696	2.837

Urban-rural	rural	.262	4.646	.031	1.299	1.024	1.648

Age (yr)	6	1					
	3	.766	22.380	.000	2.150	1.566	2.953
	4	.169	1.053	.305	1.184	.858	1.634
	5	-.009	.003	.957	.991	.714	1.376

Activity time per day in kindergarten (h)	>1.0, ≤2.0	1					
	≤0.5	.668	15.337	.000	1.950	1.396	2.723
	>0.5, ≤1.0	.636	12.616	.000	1.889	1.330	2.683
	>2.0	.199	2.118	.146	1.220	.933	1.596

Child's BMI^a^	Normal weight	1					
	Under weight	1.008	38.165	.000	2.741	1.990	3.773
	Over weight	.515	8.711	.003	1.674	1.189	2.356

Father's education level	College	1					
	Middle school	.679	17.456	.000	1.973	1.434	2.714
	High school	.413	6.987	.008	1.512	1.113	2.055

Mother's childbearing age (yr)	≥20,<35	1					
	<20	.710	15.885	.000	2.034	1.435	2.884
	≥35	-.022	.004	.950	.979	.498	1.925

Constant		-3.190	116.896	.000	.041		

^a^BMI = body mass index. Binary logistic regression analysis was used.

## Data Availability

The data used to support the findings of this study are available from the corresponding author upon request.
